# Integrative Multiomics Approach to Skin: The Sinergy between Individualised Medicine and Futuristic Precision Skin Care?

**DOI:** 10.3390/metabo14030157

**Published:** 2024-03-07

**Authors:** Angelica Dessì, Roberta Pintus, Vassilios Fanos, Alice Bosco

**Affiliations:** Neonatal Intensive Care Unit, Department of Surgical Sciences, University of Cagliari, AOU Cagliari, 09124 Cagliari, Italy; angelicadessi@unica.it (A.D.); roberta.pintus@unica.it (R.P.); vafanos@tiscali.it (V.F.)

**Keywords:** skinomics, multiomics, precision skin care, skin metabolome, system biology

## Abstract

The skin is a complex ecosystem colonized by millions of microorganisms, the skin microbiota, which are crucial in regulating not only the physiological functions of the skin but also the metabolic changes underlying the onset of skin diseases. The high microbial colonization together with a low diversity at the phylum level and a high diversity at the species level of the skin is very similar to that of the gastrointestinal tract. Moreover, there is an important communication pathway along the gut–brain–skin axis, especially associated with the modulation of neurotransmitters by the microbiota. Therefore, it is evident that the high complexity of the skin system, due not only to the genetics of the host but also to the interaction of the host with resident microbes and between microbe and microbe, requires a multi-omics approach to be deeply understood. Therefore, an integrated analysis, with high-throughput technologies, of the consequences of microbial interaction with the host through the study of gene expression (genomics and metagenomics), transcription (transcriptomics and meta-transcriptomics), and protein production (proteomics and meta-proteomics) and metabolite formation (metabolomics and lipidomics) would be useful. Although to date very few studies have integrated skin metabolomics data with at least one other ‘omics’ technology, in the future, this approach will be able to provide simple and fast tests that can be routinely applied in both clinical and cosmetic settings for the identification of numerous skin diseases and conditions. It will also be possible to create large archives of multi-omics data that can predict individual responses to pharmacological treatments and the efficacy of different cosmetic products on individual subjects by means of specific allotypes, with a view to increasingly tailor-made medicine. In this review, after analyzing the complexity of the skin ecosystem, we have highlighted the usefulness of this emerging integrated omics approach for the analysis of skin problems, starting with one of the latest ‘omics’ sciences, metabolomics, which can photograph the expression of the genome during its interaction with the environment.

## 1. Introduction

The skin, the largest organ of the human body and covers the entire external surface with an extension of approximately 1.5–2 m^2^, is crucial in managing the interface between the internal molecular processes of the organism and the external environment. In fact, its molecular composition derives not only from the host’s cells but also from the resident microbiota and from molecules coming from the environment, while its chemical composition is not yet completely defined [1,2]. Thus, the skin represents the first line of defense against lesions and microbial aggression coming from the external environment.

Some of these characteristics are in common with the gastrointestinal (GI) tract, which is also one of the largest interfaces (30 m^2^) between the host and its environment [3]. Both organs are densely colonized by the microbiota, with estimates of 10^12^ microbial cells for the skin and 10^14^ for the intestine [3], characterized by low microbial diversity at the phylum level but high diversity at the species level [3]. The presence of the microbiota is fundamental for the host as it guarantees correct development and adequate regulation of the immune system, protection from pathogens, the breakdown of metabolites, and the maintenance of an efficient barrier. In fact, the most recent studies have highlighted how dysbiosis of the skin and/or intestinal microbiota has been associated with an alteration of the immune response and also related to the development of skin diseases, such as atopic dermatitis (AD), psoriasis, acne vulgaris, dandruff and even skin cancer [3,4]. Thus, it seems clear that the role of commensal microbes in the well-being of the human body goes beyond the gastrointestinal tract, affecting other organs [4,5].

Concerning this topic, new metataxonomic and metagenomic analysis techniques, with culture-independent high-throughput sequencing technologies, such as 16S rRNA amplicon sequencing and metagenomic whole shotgun sequencing, have allowed to expand the knowledge about the microorganisms of the microbiota itself. However, despite the importance of these new acquisitions, they are not sufficient to study their effects, which are much more complex. In this context, the study of the by-products of microbial metabolism, through metabolomics, can be useful in clarifying with greater precision the high complexity of the microbe-host-environment interaction. In fact, it is common that in the presence of the same skin problem, if the skin conditions are different, even simply due to the geographical area of origin, the reactions to a dermatological product are different [6]. Therefore, following the systems biology paradigm, an effective study strategy should integrate at least the main multi-omics data from metabolomics, lipidomics, and skin microbiomics not only in an attempt to highlight potential differential biomarkers but also to evaluate the possible interaction between them, in order to verify their biological significance. From this perspective, a nascent field of study is represented by skinomics, including not only metabolomics, lipidomics, and microbiomics but also genomics, proteomics, and skin transcriptomics, which through multivariate statistical methods such as PLS-DA, PCA, support vector machine, and random forest, aims to identify the differential metabolites and substances between normal and diseased skin [6].

In this review, we highlighted the necessity, usefulness, and potential limitations of applying these emerging omics approaches to investigate the skin ecosystem, starting with the present data on the skin metabolome, a comprehensive expression of the genome in its interaction with the environment.

## 2. Skin Microbiota

### 2.1. Composition

Although the skin shares a high microbial colonization with the oral cavity and the gastrointestinal tract, it, unlike all other areas, is characterized by the greatest diversity of variables that influence its surface characteristics, together with a wide variety of cell types that interact with the present microbiota. Furthermore, the skin is not only characterized by a different and unique microbiome but also presents a notable diversity of environments, with distinct physical and chemical characteristics, which determine a high diversity of microbial populations depending on the affected area [7,8,9]. Therefore, according to some authors, when using the term “skin microbiome” it must be kept in mind that on the one hand, it distinguishes the microbes present on the skin from those present in the intestine or in the mouth but on the other hand it includes populations of microorganisms that are very different from each other depending not only on the skin site studied but also on the environment to which the individual is exposed, eliminating the definition of “biome” as a similar condition that hosts a distinct community. These considerations also determine an implicit difficulty in comparing the different studies performed on the subject [7].

Having said this premise, from the metataxonomic analyses with high-throughput sequencing technologies and independent 16s RNA culture, it is clear that the phyla making up the majority of the “normal” skin microflora coincide with the main protagonists also present in the gastrointestinal tract and in the oral cavity, i.e., Actinobacteria, Bacteroidetes, Firmicutes, and Proteobacteria, albeit in different proportions [10]. In fact, in the skin, the most abundant phyla are represented by Actinobacteria [7]. Indeed, recent work through high-throughput metagenomic sequencing has highlighted that in adults, human skin is dominated by gram-positive bacteria belonging to the genera *Staphylococcus*, *Corynebacterium*, *Enhydrobacter*, and *Micrococcus* [11]. On the other hand, culturomics studies have demonstrated the presence of gram-negative bacteria as frequent and disseminated components of the transient skin flora [12] especially, among the cultivable species, *Roseomonas mucosa*, *Pseudomonas*, *Acinetobacter* [13].

Initially, the skin surface is colonized at birth and the mode of delivery appears to impact its composition immediately, although the long-term consequences are not yet well known. Neonates born by cesarean section are predominantly colonized by commensal skin bacteria belonging to the Streptococcus, Staphylococcus, and Propionibacterium genera. Differently, in those born by vaginal birth, microorganisms common to the female urogenital tract such as *Lactobacillus*, *Prevotella*, and *Candida* prevail [14].

However, the composition of the skin microbiota is not static but evolves dynamically right from the start, increasing biodiversity during the first years of life in a site-specific manner [1,14,15,16] albeit with notable inter-individual variations [14,15].

Furthermore, a delicate period is represented by puberty, where considerable hormonal variations significantly modify the physicochemical properties of the skin surface, favoring the development of lipophilic taxa, especially *Corynebacterium* and *Propionibacterium* [14,15,16], and a decrease in Firmicutes (including *Staphylococcus* and *Streptococcus*) [16].

In adulthood, the individual skin microbiome remains relatively stable, supporting the probable establishment of mutualistic and commensal interactions not only between microbes but also between microbes and the host, involving bacterial species often considered opportunistic pathogens [1,14,17].

Moreover, as already said before, the site-specific diversification of the skin microbiota is crucial as a function of the different distribution, density, and variety of sweat glands, sebaceous glands, and hair follicles, responsible for the presence of areas of the skin with different chemical-physical properties. In detail, in particularly sebaceous skin areas, such as the face and torso, a less diversified and less rich microbiota prevails with a prevalence of *Cutibacterium* (formerly *Propionibacterium*) and partly *Staphylococcus* [7,16,18]. While, *Corynebacterium*, *Staphylococcus*, and *ß-Proteobacteria* dominate moist areas such as the armpits and the creases of the elbows and knees [7,16]. Instead, in particularly dry areas, *Micrococcus*, *Enhydrobacter*, and *Streptococcus* are also found [8,10,19,20].

The main phyla and most represented genera in a healthy adult skin microbiota are summarized in Table 1 [7,10,11,16,18,19,20].

Therefore, it is clear that the specific composition of the skin microbiome is closely related to the chemistry of a particular skin niche and specific numerous microbial and host factors which further contribute to important differences in composition at the species and strain level that may lead to distinct consequences on the host itself [16]. In fact, the most recent innovations in the field of microbiomics have highlighted how the understanding of intra-species and/or intra-population genetic heterogeneity can be crucial [16,21,22]. For example, the colonization of the same strain of *Cutibacterium acnes* is observed on multiple body sites of the same individual, while for the *Staphylococcus epidermidis* species, different strains have been highlighted, depending on the skin area involved [16,21] with variations in the pathogenetic contribution for some pathologies or skin conditions such as atopic dermatitis [21]. These data support the importance of going beyond the representative bacterial taxonomic estimate obtained by targeted sequencing of the hypervariable regions of the 16S rRNA gene and evaluating the genetic contribution of each member of the single species, investigated in terms of functional genes, through shotgun metagenomic sequencing [22].

Finally, bacterial colonization is accompanied by the presence of Archaea (Thaumarchaeota and Euryarchaeota), viruses (predominance of bacteriophages), and fungi (*Malassezia*, *Cryptococcus*, *Rhodotorula*, and *Candida*) [4].

### 2.2. Role and Alteration

The presence of this colonization is absolutely fundamental for the host, especially for the maintenance of skin homeostasis, the barrier function of the skin, and its protective role against potential pathogens and environmental aggressions [4,23]. In fact, the formation of mature keratinocytes in the stratum corneum is strictly dependent on the skin microbiota [23]. Furthermore, the presence of commensal skin microbes leads to healthy competition for nutrients and space, thus greatly influencing the growth potential of any pathogens [7,23]. In addition, microbiota produces fundamental enzymes, especially proteases, lipases, and urease which, in turn, regulate the renewal of the stratum corneum, and lipases regulate the lipid film. Furthermore, the production of sebum and free fatty acids is useful in regulating skin pH while the production of antimicrobial peptides (AMPs) is essential in protecting against potential pathogens [23]. Moreover, microbiota is involved in the regulation and communication with the immune system, the production of hormones, neurotransmitters, and cytokines, and eventually in wound repair [4,23]. As regards the cross-talk with the immune system, to date, there is a lot of evidence regarding the role of *S. epidermidis* in enhancing the host’s immunity to *S. aureus* infection thanks to the recognition implemented by keratinocytes through the Toll-like receptor 2. This appears to determine not only the increase in the expression of antimicrobial peptides but also the inhibition of the production of inflammatory cytokines, the enhancement of tight junctions in cultured keratinocytes, and the reduction of levels of inflammation in vivo, in case of injury [7].

Although the important contribution of the skin microbiota to the normal development and functioning of the immune system appears evident, the etiopathogenetic contribution of its alteration in the case of disease is not yet completely clear.

At the local level, it is now known that in the presence of particular skin pathologies, an alteration of the skin microbiota is detected; however, it has not yet been understood whether these alterations are the cause or consequence of the pathology itself.

For example, chronic inflammation of the skin in AD patients appears to cause a reduction in the presence of *Cutibacterium*, *Streptococcus*, *Acinetobacter*, and *Corynebacterium* and a consequent increase in *Staphylococcus aureus* strains, the density of which was found to be closely correlated to the severity of the disease [23]. Nonetheless, it has not yet been clarified whether the alterations of the skin barrier typical of the pathology, with different etiological causes, trigger the alterations of the skin microbiota or whether it is an initial excessive growth of certain *Staphylococcus* species that subsequently leads to the onset of the alterations typical of the disease [11]. On the contrary, a skin flora considered normal in humans, with a prevalence of *Staphylococcus epidermidis*, improves innate immunity, has anti-inflammatory effects, and helps prevent the proliferation of pathogenic bacteria [23].

In psoriasis, another inflammatory skin condition characterized by erythematous and scaly plaques that commonly occur on elbows, knees, scalp, and trunk, the definitive cause–effect relationship between disease and microbial diversity is even less solid. In fact, although detailed analysis of the skin microbiota in lesioned and non-lesioned skin of psoriasis has identified potential differences in the composition of the microbiota between skin affected by the disease and normal skin, no microorganisms unequivocally correlated to the pathogenesis of the disease have been identified yet [14]. 

Nonetheless, a microbial imbalance at the skin level can represent a significant issue not only in the case of local skin pathologies but also in the presence of other systemic inflammatory diseases, such as food allergies. In fact, the production of microbial metabolites at the skin level can have repercussions on the entire organism [23,24,25]. Specifically, as regards food allergies (FA), the presence of specific IgE towards egg whites in children suffering from AD was significantly correlated with the detection of specific IgE towards staphylococcal toxins [24,25]. This may be due to the presence of a cutaneous pathway of sensitization to IgE, which emerged thanks to studies conducted with tape stripping, which mimics the alteration of the AD-related skin barrier. Such research has in fact found that in eczematous skin exposed to allergens, there is an increase in the same epidermal alarmins involved in the immune dysregulation that characterizes AD, responsible for the hyperactivation of the Th-2 immune response [24,25]. Furthermore, exposure to particular non-pathogenic bacteria typical of rural environments, such as *Acinetobacter*, or to mothers’ germs during spontaneous childbirth, also seem to represent a protective agent against allergies, providing solid evidence of the ability of the symbiosis of the skin microbiota to control the systemic immune response of the entire organism [23].

Moreover, although the commensal microbiota is normally harmless or even beneficial to the host, some species have the ability to cause infections. This is the case of the already discussed *S. epidermidis*, often the cause of nosocomial infections of deep tissues that could be due to the presence of medical devices, such as catheters. Furthermore, bacterial species typical of the common skin microflora can infect wounds, especially chronic non-healing ones, common in diabetic patients or the elderly [14].

### 2.3. Manipulation

Given the critical role of the skin microbiota in maintaining skin homeostasis, its manipulation may be a promising approach for the treatment of numerous skin diseases [4,26,27,28]. The manipulation of the skin microbiota can be performed through different techniques, i.e., by using specific antibacterials, as in the case of trying to correct inadequate underarm odor. This approach, in addition to the possible disadvantages related to the use of antibiotics, is not exhaustive as it must be supplemented by the introduction of a healthy microbiome [4]. 

Another possibility is skin microbiome transplantation, namely the transfer of a skin microbiome from one individual into the washed and/or disinfected skin area of another person. Even in this case, there are some critical issues, including the difficulty of taking sufficient quantities of bacteria from a person’s skin. Thus, there is a need for a culture phase, which could complicate the use of this technique. It is also necessary to assess whether potential pathogenic taxa can also be transmitted [26]. 

A further strategy is skin bacteriotherapy, in which one or more pure cultures with health-promoting properties are placed on a person’s washed and/or disinfected skin area. This can be done through the application of probiotics (live and viable microorganisms), postbiotics (bacteria that are no longer viable, tindalized, or cell lysates), enzymes, or by-products of bacterial fermentation. In contrast to microbiota transplantation, skin bacteriotherapy is a scalable process that is easier to apply in an industrial context, although it has certain limitations in terms of the difficulty of grafting [4,26]. Indeed, to date, several patents have been developed focusing on various bacterial strains that could improve skin well-being, but no scientific evidence is yet available to support their efficacy [4]. 

Finally, there is a further method based on prebiotic stimulation. It is based on applying molecules with prebiotic action to the skin to stimulate the growth of specific beneficial microbes. Although this strategy does not involve a possible hyper-activation of the immune system such as the administration of bacteria, it is an indirect method with often less striking and more difficult to predict results [4]. 

## 3. The Gut-Skin Brain Axis in Health and Disease

The term gut–skin axis describes the intricate interaction between the gut and the skin, although the link between skin health and immunological responses caused by the gut microbiome is not yet fully understood [29]. It is known that it is mainly the immune system that mediates the communication between these two organs densely colonized by microorganisms, especially commensals, and that in this interaction the main role of the intestinal microbiota is to manage both systemic and local inflammation, enhancing barrier immunity [29,30]. In fact, at the intestinal level, the homeostatic balance in the host is guaranteed by the perfect balance between the “mucosal firewall”, (barrier of intestinal epithelial cells, mucus layer, T lymphocytes, IgA, and dendritic cells, DC) and the associated lymphoid tissues to the intestine, GALT. The GALT, composed of M cells, conventional lymphocytes (regulatory T lymphocytes, Tregs, helper T lymphocytes, Th, cytotoxic T lymphocytes, and IgA-producing B lymphocytes), phagocytes (DCs, mast cells, neutrophils, and macrophages), non-conventional lymphocytes (innate lymphocyte cells, ILC) and T cells (MAIT), develops thanks to complex mechanisms underlying the involvement of the intestinal microbiota [29,31]. In fact, the differentiation of Tregs specific for the commensal bacteria of the intestine, of IgA-producing B cells, and of Th17 cells occurs through the presentation of commensal antigens by the DCs. Thus, a complex balance is determined between the recognition of non-specific infections that activate the innate and adaptive immune system and tolerance towards commensals. In addition, secretory IgA exerts an important role in spatial recognition of the host tissue and intestinal microbes modulating inflammatory responses. Furthermore, the intestinal microbiota, through the production of short-chain fatty acids, SCFAs, in particular butyrate, reduces the permeability of the intestinal barrier and improves its integrity [29].

Therefore, in case of disruption of intestinal integrity and/or an imbalance of its microbial communities can affect skin homeostasis, influencing, for example, the severity of a skin pathology such as acne. These data are in agreement with the contribution of the intestinal microbiota in the regulation of systemic inflammation, oxidative stress but also emotional changes [32,33,34]. In fact, the brain is also involved in this complex interaction between the intestine and skin, through neurotransmitters (mainly GABA or gamma-aminobutyric acid, acetylcholine, dopamine), SCFAs, secondary bile acids, and tryptophan metabolites [32,34]. The concept of the gut–brain–skin axis was first proposed in 1930 by two dermatologists, Stokes and Pillsbury, who hypothesized a contribution of the gastrointestinal tract in mediating the influence of emotional and nervous states on the skin [29]. These hypotheses were confirmed in subsequent studies in which the role of gastrointestinal tract bacteria in regulating some connections between emotional states and inflammatory skin conditions was demonstrated. Indeed, it is now accepted by the scientific community that chronic skin inflammation and mental health disorders are often co-morbid [34].

**Figure 1:**
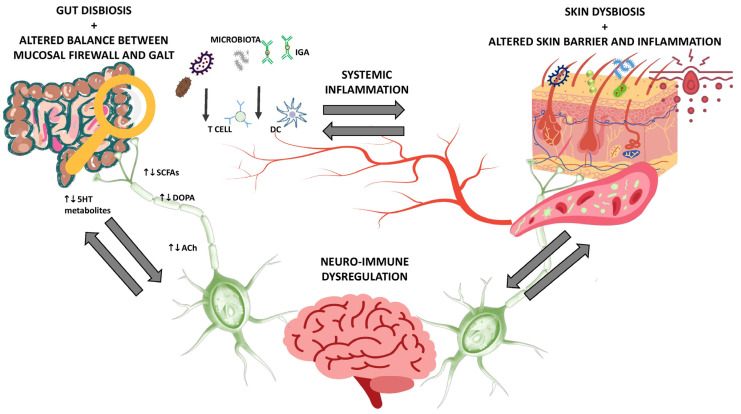
Gut–skin–brain axis. Abbreviations: 5HT, 5-hydroxytryptamine; SCFAs, short chain fatty acids; DOPA, dopamine; ACh, acetylCholine; IGA, immunoglobulin A; DC, dendritic cells.

It is also noteworthy that some of these microbially derived neurotransmitters appear to regulate the function of immune cells in the host via the nervous system. In fact, it is through the neuroimmune and neuroendocrine systems that the skin-intestine-brain cross-talk is made possible, in which some neuroactive molecules of microbial origin, such as GABA, 5-hydroxytryptamine (5-HT), norepinephrine, and dopamine are the endogenous signals for the central nervous system [34]. Although it is not yet fully understood how these metabolites elicit a response in the host, it seems that these molecules can not only regulate the homeostatic balance at the skin level, altering the integrity of the skin barrier and epidermal differentiation, but also have systemic generalized effects [32,34]. Nonetheless, further specific experimental studies are certainly necessary to fully confirm these hypotheses [34]. The main mechanisms of the skin-gut-brain axis are summarized in Figure 1.

Regarding acne vulgaris, the potential pathways involved in the gut–brain–skin axis have been highlighted by the work conducted by Bowe et al. [33]. They hypothesized that initial psychological distress, alone or in combination with a diet rich in processed, high-fat, and low-fiber foods, may cause alterations in intestinal function and dysbiosis. This determines greater intestinal permeability, causing endotoxemia, which in turn is responsible for an increase in oxidative stress and inflammation together with a decrease in insulin sensitivity. This condition, in genetically predisposed subjects, can increase the production of sebum, exacerbating acne and eventually increasing psychological distress.

In the case of AD, there are several potential pathways involved in the gut–brain–skin axis. First of all, the first studies with independent culture methods had already highlighted an atopy-related dysbiosis, with a reduction in the ratio between Bifidobacteria and Clostridia in atopic children. The authors highlighted how such differences in neonatal intestinal microflora preceded the development of atopy, hypothesizing a crucial role of the balance of indigenous intestinal bacteria for the maturation of human immunity in a non-atopic manner [35]. Further confirmation of this hypothesis is the KOALA Birth Cohort Study [36] in which a dysbiosis was observed prior to the development of atopy in which the increase in *E. coli* was associated only with dermatitis, while the increase in *C. difficile* was related to all atopic outcomes, including recurrent wheezing and allergic sensitization. It should be underlined that these alterations, as a whole, contribute to an increase in the general inflammatory state [3]. Moreover, in atopic subjects, lower levels of Bifidobacterium and higher frequency of *Staphylococcus* [37]. Furthermore, atopy-related CD4+ T-cell dysfunction, according to some authors [38], appears to be associated with a lower relative abundance of *Akkermansia* and *Faecalibacterium*, a higher relative abundance of particular fungi such as *Candida* and *Rhodotorula*, and a fecal metabolome characterized by pro-inflammatory metabolites.

In addition, a particular intraspecies intestinal dysbiosis of *Faecalibacterium prausnitzii* has been observed in patients with AD by Song et al. [39]. Indeed, the analysis of the OTUs of *F. prausnitzii* found a significant increase in one of the clades, F06, in the microbiota of patients with AD. This alteration was common at all ages, but more evident in the group of children under 1 year of age. This is very interesting considering the typical age of onset of this pathology and leading to the hypothesis of its involvement in its onset. In fact, they highlighted how the balance between the subspecies of *F. prausnitzii* can also determine changes in the production of SCFAs. This is in agreement with the data emerging from the analysis of the microbiome, which show a high presence of genes involved in the metabolism of some nutrients deriving from the damaged intestinal epithelium, supporting a possible increase in inflammation in the epithelium itself. Therefore, according to the authors, in the presence of such mucosal damage, perhaps triggered by the intraspecies dysbiosis of *F. prausnitzii* or by other unknown causes, an overgrowth of various pathobionts or auxotrophs may occur which further fuels the dysbiosis of *F. prausnitzii*, establishing a feedback loop between dysbiosis itself and the dysregulation of inflammation of the intestinal epithelium. The resulting increased intestinal permeability may lead to the passage of undigested foods, toxins, and pathogenic microbes into the systemic circulation and be responsible for aberrant TH2-type immune responses to such foreign substances, leading to increased inflammation in the deep layers of the skin [39].

It is thus evident that, overall, this intestinal dysbiosis may result in the alteration of the metabolites and neurotransmitters produced, and the altered immune cells can reach the circulatory system influencing the onset and progression of skin pathologies such as AD [29]. In this regard, in a mouse model of AD, the possible role of GABA in the attenuation of skin lesions was demonstrated, favoring the predominance of type 1 T helper cells (Th1) to the detriment of T helper cells type 2 (Th2), supporting how an alteration in the production of microbial metabolites can affect the gut–brain–skin axis [40].

In addition to possible internal stressors, such as infections or dysbiosis, there are external stress factors, such as psychological ones, which contribute to compromising the skin barrier and favoring a shift in immunity towards an allergic response of type-2 helper T cells, through the involvement of different neurotransmitters. It is in fact now known, thanks to psychoneuroimmunology, that the skin represents not only the target of stress mediators but is also an active producer of them, with important repercussions in many skin diseases such as AD [41,42].

Furthermore, the gut–brain–skin axis is also involved in psoriasis. In fact, the release of neurotransmitters by the gut microbiota, mediating communication between the immune system and the nervous system, can lead to an inappropriate activation of various immune pathways leading to an increased production of pro-inflammatory cytokines, which are typical features of this disease [34]. 

Nevertheless, although the composition of the intestinal microbiota and their association with psoriasis are not yet fully clarified, a significant decrease in *A. muciniphila*, a species that seems to be able to strengthen the integrity of the intestinal epithelium and protect against systemic inflammatory diseases [43], together with a reduced relative abundance of *Ruminococcus* and *Pseudobutyrivibrio* have been observed [44]. However, in psoriasis, there is currently no evidence regarding specific neurotransmitters capable of treating the pathology but research in this field is still very active given the growing evidence regarding the role of immunomodulation by neurotransmitters of microbial origin [34].

## 4. Integrative Multiomics Approach to Skin

The application of systems biology allows studying the functional variation of molecules and their interactions in cells, tissues, organs, and organisms, using systematic quantification methods thanks to the application of mathematical models [6]. These considerations are fully reflected in the study of the microbiome, now considered a leading player in maintaining the state of health of human beings. Indeed, although to date advanced metagenomics techniques have led to notable progress in the classification of the microbiome, its application does not allow us to fully understand yet either the complex interactions within the microbiota itself or those with the host, such as changes in the gene expression and the resulting metabolic by-products. Thus, it is still insufficient to describe the pathogenesis of numerous skin diseases and/or problems [5]. Therefore, it is clear that it is necessary to consider changes in gene expression, metabolite production, and microbial interactions with the host using the integration of multiple omics data, such as genomics (and/or metagenomics), transcriptomics (and/or meta-transcriptomics), proteomics (and/or meta-proteomics) and metabolomics [5]. The definition of the main omics technologies and their applications are summarized in Table 2. 

Through metagenomics it is possible to identify the taxonomic composition of the microbiota together with the characterization of the relative abundances of the taxa and the description of the functional contribution of each taxon, also highlighting intra-species and/or intra-population genetic heterogeneity. In fact, if on the one hand, the targeted sequencing techniques of the hypervariable regions of the 16S rRNA gene allow a representative bacterial taxonomic estimate, it is only through shotgun metagenomic sequencing that it is possible to evaluate the genetic contribution of each member of the community investigated in terms of functional genes [22].

**Figure 2:**
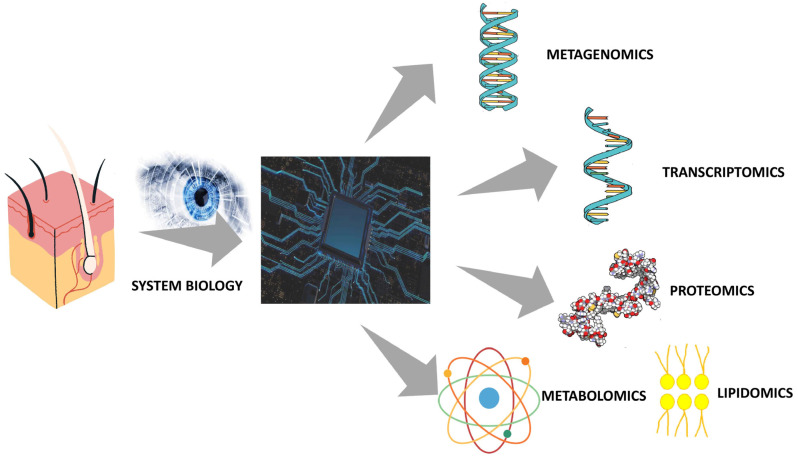
System biology approach to skin.

The integration of these data with meta-transcriptomics, meaning the study of gene expression at the community level, makes it possible to understand the expression of the sequences highlighted by metagenomics, identifying the genes actively expressed in complex bacterial communities in order to understand the metabolic processes active in microbial communities in different environments but also its interactions with the host and the functional alterations that determine the transition from a healthy microbiome to a dysbiosis [5,45]. Meta-proteinomics allows the entire protein complement of the microbiota to be characterized on a large scale [46], highlighting the expression of active functional pathways with greater precision. In fact, it is possible to obtain more precise data, in terms of protein production, compared to what is observed with meta-transcriptomics that does not highlight the precise correlation between the production rate and the concentration and stability of the proteins actually present [5]. Nevertheless, it is one of the most recent “omics” sciences, metabolomics, that stands out among others for its high predictive capacity of the phenotype. In fact, through the metabolome, it is possible to photograph the genome expression during its interaction with the environment and therefore investigate the metabolic state of an organism under certain conditions [38]. This is achieved thanks to the systematic study of the complete set of metabolites (metabolome) present in given biofluids, cells, or organisms [47], allowing a distinction among those of microbial origins, signaling molecules, and those produced by the host [5]. This could overcome some limitations of other omics by providing a precise snapshot of phenotypic expression but complementarity with other investigation techniques remains fundamental as it is currently not possible to translate all the data into a significant biological hypothesis [5]. An important branch of metabolomics is represented by lipidomics, meaning the complete and systematic analysis of a particular type of body metabolite, lipids. Given the delicate role of skin lipids in skin homeostasis, this method can certainly broaden knowledge in this field. In fact, a change in the content and composition of skin lipids has significant repercussions on the metabolism and inflammation of skin cells, influencing the regulation of cell proliferation, the induction of cell differentiation, and the initiation of apoptosis. Furthermore, there is a very close correlation between the skin lipid composition and the skin microbiota [48]. The application of systems biology to the study of the skin is schematized in Figure 2.

Although the remarkable advances in the field of omics science have implemented the understanding of different disease processes, and identifying complex molecular networks, most of the results appear disconnected and divergent, making their use still too limited [49]. 

First of all, a key point in making the information generated by the integration of these omics data usable is both the high number of different variants and the identification of differences through data normalization and dimensionality reduction [50]. This is possible through a multistage approach with initial emphasis or a multimodal approach. In the former case, various types of omics data are initially incorporated into the target model creation procedure, followed by the application of the molecular data from the different omics to the target model individually to produce the final model results. In contrast, the multimodal approach involves the integration of multiple omics profiles in a simultaneous analysis, with an analytical model based on machine learning [50].

In addition, multi-omics platforms are being created to understand complex molecular networks using existing data, such as the PlatOMICs devised by Brandão et al. [49]. The use of such platforms may prove useful in integrating and re-analyzing information in search of molecular interactions involved in the pathogenesis of numerous skin diseases to develop and support more precise hypotheses. This software is necessary to complete various databases, such as the Human Metabolome Database that provides specific molecular profiles [50].

However, multi-omics analysis should be integrated at an early stage of study design, rather than being a post-hoc process after data production [51]. 

In this regard, to date, only eight studies have integrated skin metabolomics data with at least one other “omics” technology (summarized in Table 3) [52,53,54,55,56,57,58,59].

Among these, only three investigations included the study of the skin microbiome in a multiomics analysis of the skin. Indeed, recent evidence would even seem to suggest the need to integrate metagenomic profiling with culturomics [6], namely the use of different culture conditions together with identification by matrix-assisted laser desorption mass spectrometry (MALDI-TOF MS), for a further expansion of knowledge on the bacterial repertoire [60]. Indeed, considerable variation at the bacterial strain level in the local microenvironment has recently emerged, with lineages (sets of colonies separated by <100 mutations) specific to the individual skin pore [61].

One of the most recent investigations was conducted by Ashrafi et al. [52]. This is an exploratory temporal profiling study of the skin microbiome and metabolome aimed at clarifying the wound healing process through non-invasive methods. Metabolites analyzed through gas chromatography-mass spectrometry (GC-MS) and microbiome analysis, with 16sRNA sequencing, highlighted the temporal and dynamic nature of the metabolome and wound microbiome. These results may allow us to identify a potential signature of the microbiome and metabolome of the various phases of skin healing through the detection of possible biomarkers corresponding to the different times of tissue repair.

The study by Emmert et al. [53] is the first to highlight site-specific lipid alterations and correlations with the skin microbiome in AD. In fact, they observed a significant variation in skin lipid composition not only in different body areas but also between patients with AD and healthy controls. Specifically, they detected a significantly higher concentration of the ceramide species NS in AD patients compared to healthy volunteers. They also found that this alteration was greater in AD patients with a filaggrin (FLG) mutation than in AD patients without an FLG mutation. Finally, through the correlation analysis of skin lipid alterations with the microbiome, it was possible to highlight a positive correlation between Staphylococcus colonization in AD and the ceramide subspecies AS, ADS, NS, and NDS.

Instead, Kuehne et al. [54] conducted an integrative metabolomics and transcriptomics investigation to identify metabolic alterations in the aged skin of humans in vivo in order to identify possible topical treatments that can potentially reverse or alleviate age-related skin deterioration. The multi-omics approach identified transcription-induced alterations in metabolism during aging, such as the alteration of the activity of higher glycolysis and glycerol-lipid biosynthesis or the decrease in the biosynthesis of proteins and polyamines. The authors hypothesized a close correlation between the highlighted alterations and cell signaling, the function of the epidermal barrier, and the structure and morphology of the skin. Therefore, they concluded that these advances in understanding the impact of metabolism on age-related alterations in skin function may lead to improved skin care treatments.

Furthermore, Marathe et al. [55] conducted a multi-omics analysis to better understand the role of IL-9 in the metabolic reprogramming of human primary keratinocytes (KCs). Through high-throughput quantitative proteomics, IL-9-induced alteration of the electron transport chain in primary human KCs was discovered. Instead, metabolomic investigation with nuclear magnetic resonance (MRI) highlighted the IL-9 induced reduction effect, on the production of intermediates of the tricarboxylic acid cycle in primary human KCs. From the integration of all multi-omics data, the authors concluded that IL-9 is responsible for numerous effects on carbohydrate metabolism, particularly in relation to the glycolytic switch, revealing its role as the main regulator of metabolic reprogramming and KC survival.

Studies with a broader multi-omics approach, which integrated several omics analyses were conducted by Acharjee et al. [56], Zhou et al. [57], Mirsa et al. [58], and Tilton et al. [59] as well.

Acharjee et al. [56], given the importance of the multi-omics approach to understand the pathophysiological mechanisms of AD and its clinical manifestations, conducted an integrated analysis of multi-omics data from multiple cohorts in order to identify candidate genes relevant to AD and to associate them with particular metabolites and miRNAs. They first highlighted, thanks to four different datasets on the transcriptome, some robust hub genes that can be used as gene “signatures” concerning crucial pathogenetic elements in AD. Then, they detect the presence of specific metabolomic pathways associated with particular gene signatures, demonstrating the existence of characteristic associations between genes, metabolites, and miRNAs in AD.

Instead, Zhou et al. [57] through a multi-omics investigation, including microRNAomics, proteomics, and metabolomics, investigated the mechanism underlying skin tumorigenesis induced by prolonged exposure to arsenic. This approach highlighted the presence, in transformed cells induced by arsenic, of alterations in the profiles of miRNAs, proteins, and metabolites. These results improved the knowledge regarding the malignant transformation of cells induced by arsenic and to identification of potential early biomarkers for cutaneous squamous cell carcinoma induced by this exposure.

Moreover, Misra et al. [58] employed a multi-omics analysis using metagenomics, proteomics, and metabolomics to decipher the molecular link between chronic pollution exposure and human skin dysfunction. They conducted an untargeted metabolomic analysis on skin samples to characterize pollution-dependent biochemical events, followed by a targeted proteomic analysis and 16S and ITS amplicon sequencing to define the microbiome. The data thus obtained were subjected to a block-structured multivariate analysis in order to create a molecular map consisting of potential multi-omics signatures that correlate with the presence of skin dysfunctions in individuals living in a polluted environment. The multi-omics approach highlighted macromolecular skin alterations due to pollution which could manifest as clinical signs of premature skin pigmentation and/or other blemishes.

A complex pioneering study was performed by Tilton et al. [59] to investigate how biological systems respond to subtle perturbations in their environment following exposure to low-dose radiation. They used a full-thickness 3-D human skin in vitro model to examine, across transcriptomic, proteomic, phospho-proteomic, and metabolomic platforms, the temporal response of the dermal and epidermal layers to 10 cGy of X-rays. Through the bioinformatic analysis of each dataset, the authors were able to independently highlight potential signaling mechanisms influenced by radiation. However, it is only thanks to data integration that it has been possible to expand information on the mechanisms regulating low-dose responses in human tissues. In fact, the bottom-up approach, namely the integration of the pathways, has allowed the identification of molecular responses and common pathways to low-dose radiation (oxidative stress, nitric oxide signaling, and transcriptional regulation through the SP1 factor), not highlighted by the individual datasets, supporting the importance of the multi-omics approach to describe in detail the response mechanisms of complex biological systems.

Finally, although multi-omics approaches integrate data obtained from different omics techniques to understand their interrelation and combined influence on disease processes, some common problems and limitations remain. These include experimental and technical variations (so-called batch effects) and biological variations. In fact, with the exception of the genetic profile, which is identical between tissues and cell types, all other omics profiles (i.e., transcriptome, epigenome, proteome, and metabolome) vary between tissues. Nevertheless, in recent years, the underlying high-throughput technologies, such as sequencing and mass spectrometry, have become more sensitive, accurate, and accessible making the multi-omics approach a promising and powerful tool [51].

## 5. The Future of Skin Care: Between Individualized Medicine and Precision Cosmetics

For years, skincare meant the application of traditional cosmetic products, designed according to a subjective classification of skin type obtained through different questionnaire models. This approach, combined with the observation of the effectiveness of the applied treatment, proved to be simple and rapid but excessively correlated to a subjective interpretation, of the cultural background of the enrolled subjects and often characterized by poor correlation between the declared skin types and the corresponding results of the physical examination [6].

Effectiveness-based skin care was therefore developed, based on the use of a specific treatment only after a careful analysis of the skin was carried out thanks to the combination of a non-invasive examination aimed at detecting important biophysical parameters of the skin, together with skin imaging. This method, thanks to innovative non-invasive equipment, is able to detect some important skin parameters such as pH, transepidermal water loss (TEWL), hydration of the stratum corneum (SCH) and its thickness, the content of sebum and skin elasticity [6].

However, despite considerable progress in this field, one-way studies are not able to define all skin problems. In fact, as widely discussed, the scientific community performed broader studies, based on genomics, proteomics, transcriptomics, and metabolomics (and lipidomics) of the skin, giving rise to an innovative field of study: skinomics. It has been widely described how this method offers the advantage of a more in-depth understanding of the metabolic states of various skin types in different conditions, also allowing the identification of potential biomarkers of different skin types [6,62]. In fact, through multi-omics integration, it could be possible to analyze not only the metabolic pathways inherent to particular genes, proteins, metabolites, and the microbiome but also the network of their eventual interactions, thanks to multivariate statistical methods. It is therefore possible to validate the biological function of differential substances through the integration of multi-omics data, although statistical significance is not synonymous with biological significance [6].

In this regard, already a decade ago, a paper published in *Nature* [63] discussed how the rise of modern technologies in skin care, including genomics, could allow the creation of personalized skin care products. Nevertheless, even in this case, it was highlighted that genetics is only a starting point that requires broader studies to explore the biology of the skin as a whole, in an attempt to propose cosmetic products based on science that actually improve skin conditions. Thus, the multi-omics approach can represent the foundation for the personalization of skin care, both in pathological conditions and for pure cosmetic ambitions. From this perspective, some authors [6] hypothesize that once the differential biomarkers have been highlighted, the skin can be normalized through the administration of deficient substances or other ingredients capable of modulating the expression of altered metabolites. Although this goal is still far away, there are the first studies aimed at finding an increasingly tailor-made skin care product.

In this respect, Seité et al. [64], starting from recent data on the delicate role of skin dysbiosis in AD, demonstrated the effectiveness of a specific emollient containing a biomass of non-pathogenic bacteria of *Vitreoscilla filiformis*, in normalizing the skin microbiota and in significantly reducing the number and severity of flare-ups of the disease, compared to another emollient. Also, Yang et al. [65] tried to improve the tailor-made cosmetic research. In their study, the application of an optimal lipid mixture containing cholesterol, ceramides, and two fatty acids, palmitate and linoleate (4.3:2.3:1:1.8), significantly improved the barrier function of the stratum corneum after it had been altered with detergents or tape-stripping, supporting the usefulness of replenishing these lipids through the direct application of the missing lipids in the treatment of some skin problems both of a pathological nature and due to aging. However, this treatment showed no effect on skin treated with some detergents such as sodium dodecyl sulfate (SDS), probably due to the protein denaturation of the surfactants and their penetration into the deeper nucleated layers of the epidermis. This confirms the results from other studies which have highlighted how the incorporation of non-deficient stratum corneum lipids could even compromise the skin barrier function. The importance of a personalized treatment therefore emerges even in the simple replenishment of the lipids of the stratum corneum, in such a way as to respond to the real metabolic alterations of the skin [66].

In addition, as emerges from the review by Jiang et al. [6], the creation of a particular algorithm, by Touumazou et al. [67], suitable for evaluating the ideal cosmetic product according to the individual subject, through the integration of data on single nucleotide polymorphism (SNP) of individuals. Specifically, it consists of searching for the presence of SNPs on a sample of an individual’s genetic material, in a predefined set of single nucleotide positions. The presence or absence of a mononucleotide polymorphism allows the identification of one or more parameters for each localization which are then used to determine a score of the individual product, indicative of its suitability for the individual [67].

From this perspective, an important contribution may also come from the expansion in the use of DNA microarrays for transcriptional profiling in the field of dermatology. The most advanced studies of such technology to date have been conducted on melanomas, one of the most aggressive human cancers. Indeed, DNA microarrays have already proven effective in comparing metastatic and non-metastatic melanomas, in integrating transcriptional and genetic data of melanoma, and in envisaging a broad use for classification and sub-classification of neoplasms, severity assessment, and prognosis. This will make it possible not only to type and subtype carcinomas and melanomas but also to provide increasingly specific and personalized treatments, to monitor the efficacy of therapy and to optimize the timing of diagnosis. In addition, similar types of microarrays could be used to characterize the skin microbiome in order to positively improve its interactions with the host. Thus, the potential for dermatology to harness the power of microarrays quickly and efficiently can be seen by envisaging them as a means of improving both the understanding of healthy and pathological processes in the skin, including neoplasms, inflammatory diseases, and wound healing and for cosmetic dermatology with a view to increasingly personalize skin care products [6,68].

## 6. Conclusions

The important role of the skin metabolome both for the characterization of the physiological functions of the skin and for the identification of the metabolic changes underlying the onset of skin pathologies is now widely recognized. However, probably only through a multi-omics approach will it be possible to decipher the high complexity of the skin system, deriving not only from the genetics of the host but also from the interaction of the host with resident microbes and between microbe and microbe.

A further element of complexity is provided by the exposome, the totality of exposures coming from different internal and external sources, including chemical and biological agents, starting from conception and throughout life, recently attributed as potentially responsible for the alteration of the barrier cutaneous [69,70].

Despite the progress of analytical techniques in the complete quantification of the skin metabolome and the low invasiveness of the techniques used, the goal of translating these data into a significant biological context is still far away.

Although little data is still available today, thanks to the expansion of systems biology, in the future it will be possible to create large multi-omics data archives that, through specific algorithms, can predict both the specific responses to pharmacological treatments and the effectiveness of the different cosmetic products on individuals. 

In this regard, microarray technology, which is still too expensive and not easy to use today, could be part of the routine in dermatology examinations in the future, thanks to modern software capable of processing data and integrating them with metabolomic profiling. In fact, the ability of microarrays to analyze all possible types of skin lesions will allow a broadening of knowledge and an improvement in therapies for skin problems together with considerable progress in the field of prevention, thus also favoring cosmetic dermatology.

Thus, the expansion of skinomics may in the future provide simple and fast routine application tests in both clinical and cosmetic settings for the identification of numerous skin diseases and conditions.

## Figures and Tables

**Table 1:** The main phyla and most represented genera of healthy adult skin microbiota.

Phylum	Genus
Actinobacteria	*Corynebacterium*, *Cutibacterium*, *Micrococcus*
Firmicutes	*Staphylococcus*
Bacteroidetes	*Flavobacteriales*
Proteobacteria	*Enhydrobacter*, *ß-Proteobacteria*

**Table 2:** The main omics technologies and their applications.

Omics Technique	Definition	Application
(meta) Genomics/microbiomics	Culture-free analysis of the genetic heritage of an individual or a group of micro-organisms (meta) through innovative DNA extraction and replication techniques	Genotype detection and characterization of a microbial community
(meta) Transcriptomics	Sequencing of RNA (both mRNA and rRNA) present in a host’s cell or in a microbial community (meta)	Determination of the gene expression level and more in-depth knowledge of the active metabolic pathways
(meta) Proteomics	Protein abundances measurement and determination of protein species in the host’s cell or in the microbial community (meta)	Accurate analysis of the expression of functional active pathways
Metabolomics	Detection of host metabolites and by-products of microbial activity	Accurate snapshot of phenotypic expression and a deeper understanding of microbial communication

**Table 3:** Skin’s multiomics studies.

Authors/Years	Omics Technologies	Bio-Specimens	Technique	Results
Ashrafi et al. [52] 2020	Metabolomic and metagenomic	Wound tissue	GC-MS 16sRNA amplicon	Temporal and dynamic acute wound metabolome and microbiome for identification of possible bio-markers that correspond to wound healing processes
Emmert et al. [53] 2020	Lipidomics and metagenomic	Skin (by strip tape)	SFC-MS/MS 16sRNA amplicon	Cutaneous lipid composition alterations between body sites and correlations with the skin microbiome in AD, including in relation to FLG mutation
Kuehne et al. [54] 2017	Metabolomic and transcriptomic	Epidermal tissue	QTOF−MS	Metabolic adaptations and its transcriptional regulation during human skin’s aging
Marathe et al. [55] 2021	Metabolomic and proteomic	Epidermal tissue	LC-MS/MS ^1^H NMR	IL-9′s role as the main regulator of metabolic reprogramming and survival of KCs
Acharjee et al. [56] 2021	Metabolomic, transcriptomic, and microRNAomic	Epidermal tissue Blood Serum	LC-MS targeted Microarray	Characteristic associations between genes, metabolites, and miRNAs in AD
Zhou et al. [57] 2017	Metabolomic, proteomic and microRNAomic	Cultured Human keratinocyte HaCaT cells	UPLC/Q-TOF MS 2D-PAGE -MS Q-RT-PCR Microarray	Alterations in miRNA, protein, and metabolite profiles in arsenic-induced transformed cells, identifying potential early biomarkers for squamous cell carcinoma of the skin induced by arsenic exposure
Mirsa et al. [58] 2021	Metabolomic, proteomic, and metagenomic	skin (by strip tape and sterile cotton-tipped dry swabs) and hair samples	UPLC-MS/MS HILIC/UPLC-MS/MS MRM/SRM GC–MS/MS LC–MS/MS 16sRNA and ITS1 amplicon	Macromolecular skin changes due to pollution could manifest as clinical signs of early skin pigmentation and/or other blemishes
Tilton et al. [59] 2015	Metabolomic, transcriptomic, proteomic and phosphor-proteomic	In vitro 3D full-thickness human skin organotypic cultures	LC-MS GC-MS microarray	Identification of molecular responses and common pathways to low-dose radiation not highlighted by individual data sets, describing in detail the response mechanisms of complex biological systems

Abbreviations: GC-MS, gas chromatography–mass spectrometry; SFC-MS/MS, supercritical fluid chromatography-tandem mass spectrometry; MS-MS, tandem mass spectrometry; QTOF-MS, hybrid quadrupole time-of-flight-mass spectrometry; LC-MS/MS, liquid chromatography-tandem mass spectrometry; UPLC/QTOF-MS, ultra-performance liquid chromatography/ hybrid quadrupole time-of-flight-mass spectrometry; 2D-PAGE MS, two-dimensional polyacrylamide gel electrophoresis-mass spectrometry; Q-RT-PCR, real-time quantitative reverse transcription PCR; LC-MS, liquid chromatography-mass spectrometry; ^1^H-NMR, proton nuclear magnetic resonance; UPLC-MS/MS, ultra-performance liquid chromatography-tandem mass spectrometry; HILIC-UPLC-MS-MS, ultra-high-performance hydrophilic liquid chromatography-ultra performance liquid chromatography-tandem mass spectrometry; MRM/SRM, multiple reaction monitoring/selected reaction monitoring; AD, atopic dermatitis; FLG, filaggrin; IL-9, interleukin 9; KCs, human primary keratinocytes.

## Data Availability

Not applicable.
